# Massage and Mechano-Therapeutics at the Front: The Importance of Hastening Convalescence

**Published:** 1915-03-27

**Authors:** 


### MASSAGE AND MECHANO-THERAPEUTICS AT THE FRONT.

### The Importance of Hastening Convalescence.

The value of any therapeutic measure is, at least in a rapid judgment, usually presented in absolute terms either of affirmation or negation. Such-and-such an agent or method, we are told, is a remedy in such-and-such a disease; or, alternatively, the record runs that the curative value attributed to this remedy is without foundation. Statements of this order are frequent in popular works on medicine, nor are they altogether absent from writings which pretend to compass a more scientific audience. To support with confidence many of these statements is quite impossible, and it is often equally impossible to challenge them with success. They are made largely with the conviction that the measures proposed are not harmful, that they may perchance act in the right direction, and that experience seems to show that they harmonise with the tendency of many diseased processes to subside when rest and other favouring conditions are established. There is, however, another aspect to the influence of remedial agencies other than what may be called the " kill or cure " influence. This is the economic value of such agencies as forces tending to shorten convalescence, and to hasten the return of the worker to his position in the general scheme of things. Here is only one of the numerous issues which are started by proposals to use particular methods and agents in the treatment of disease. It may, perhaps, be allowed that a particular proposal may be useful under a great emergency, and yet its general adoption or persistence may be condemned as capable of interfering with recovery. Take, for example, the history of the controversy on the therapeutic value of alcohol. Among its many features the economic aspect has not been the least prominent, and even yet, while some advise it as facilitating recovery, others condemn it as delaying convalescence. Far be it from us to run the risks which attach to peacemakers who venture into this camp; we are content rather to utilise for the time being both of the factions, for each lays stress on the point now before us—namely, the necessity of regarding the treatment of disease in its economic relations. It is not only that gain accrues from the saving of life; it accrues also from influences which hasten recovery and promote complete and rapid restoration of function. Time saved from sickness is time added to the national account.

The circumstances of the moment add emphasis to the economic significance of illness and to the value of an abbreviation of convalescence. Never was there a moment when each unit in the national life was more important to the country as a whole. What is subtracted when so much is needed is correspondingly serious, and hence wounded soldiers and ailing workmen are not mere objects of sympathy. They are, indeed, sources of national weakness, and whatever helps to restore them to activity is a contribution to the total measure of national force. Obviously this is a sphere of influence in which medical activities must take a prominent place. Indeed, there seems a necessity to admit that, turn where you will, in the present war it is impossible to escape the doctors. Whether you wish to prepare men for the field, or to keep them fit while they are there, or to mend them when broken in the wars, the doctor seems to be an omnipresent necessity.

In this last-mentioned respect much interest may be found in an article communicated to the *Times* by a medical correspondent, and descriptive of some methods in operation at the Hôpital Militaire at Calais, under the direction of Professor Germontprez, of Lille. The problem is to hasten the restoration of joint function in cases in which stiffness has followed the immobility practised to secure the healing of fractures and other injuries. Mechano-therapeutics is by no means a new department of treatment, though it has been developed much more fully in certain Continental countries than in Britain. Probably advocates overstate its value, and contemners under-estimate it. But beyond question it has much utility in the cases to which reference has just been made. The force necessary to establish the natural excursion of the parts of a joint in which bones are bound together by adhesions can, by means of suitable apparatus, be applied with the utmost gentleness, and, what is most important, can be graded by exact measurement. Little by little in such conditions is the only safe way to freedom, and while in individual instances the violent breaking down of adhesions by main force may be necessary, the milder method is, unless circumstances forbid it, much to be preferred. A great surgeon once wrote an article on " Cases which Bone-setters Cure," and helped by it to free the profession from the haunting fear of the risks which might attend the breaking-down of adhesions in the neighbourhood of joints. To-day we have, perhaps, more elaborate and less crude methods of securing this end, though the principle remains unaffected. Exercises and apparatus can readily be devised to secure that the patient gradually practises the movement which it is desired to restore, and a general endeavour is made to add the tonic influence of personal interest, so that the patient feels he is actively co-operating in his own cure. Methods of this order, associated with the practice of massage, have a large field of opportunity in dealing with many of the injuries from which our soldiers are suffering, and it is satisfactory to know that in some measure they are available under skilled and responsible direction. We should be glad to know that there were more such opportunities in this country. Without them it is much to be feared that many a man who might have returned to the battle, or at least regained a capacity for civil employment, will remain crippled. Apart from the war, there are not a few quarters in which it has yet to be learned that the expenses of efficient medical and surgical treatment for the people can be justified as the practice of national economy.

